# Stereotactic radiosurgery treatment of pediatric arteriovenous malformations: a PRISMA systematic review and meta-analysis

**DOI:** 10.1007/s00381-025-06835-z

**Published:** 2025-05-23

**Authors:** Garrett W. Thrash, Riley Ethan Evans, Yifei Sun, Anne C. Roberts, Cameron Derryberry, Andrew T. Hale, Somnath Das, Hunter Boudreau, Jordan A. George, Travis J. Atchley, Jeffrey P. Blount, Brandon G. Rocque, James M. Johnston, Jesse G. Jones

**Affiliations:** 1 Heersink School of Medicine, University of Alabama at Birmingham, FOT Suite, 1720 2nd Ave S, Birmingham, AL 35294 USA; 2 Department of Neurosurgery, University of Alabama at Birmingham, Birmingham, AL USA; 3 Division of Pediatric Neurosurgery, Department of Neurosurgery, University of Alabama at Birmingham, Birmingham, AL USA; 4 Department of Diagnostic Radiology, University of Alabama at Birmingham, Birmingham, AL USA

**Keywords:** Stereotactic radiosurgery treatment, Pediatric arteriovenous malformations, International Stereotactic Radiosurgery Society

## Abstract

**Background:**

Stereotactic radiosurgery (SRS) is considered a safe definitive treatment for pediatric arteriovenous malformations (AVMs) upon indicated presentations. There are no published guidelines by the International Stereotactic Radiosurgery Society (ISRS) detailed with indications or characteristics that warrant SRS, other than the guideline that SRS is a safe and efficacious treatment for pediatric AVMs. SRS is performed using either Gamma Knife (GK) or Linear Accelerator (LINAC). This systematic review aims to uncover treatment, differences in GK and LINAC outcomes, and AVM characteristics that lead to high obliteration rates and suggest future studies to determine treatment decisions, raise obliteration rates, and lower complication rates in SRS treatment of pediatric AVMs.

**Methods:**

We performed a systematic review according to PRISMA guidelines across PubMed, Embase, and SCOPUS utilizing search terms related to pediatric patients, AVMS, and SRS. We collected data from the 32 full-text studies and 4 abstracts that met inclusion criteria. Subsequent pooled analysis was performed on GK vs LINAC obliteration rates, followed by sub-cohort analysis of all SRS patients with hemorrhagic presentation, Spetzler-Martin (SM) Grade, and prior procedure and their effect on obliteration rates.

**Results:**

The 36 studies reported 3425 patients, with a slight male preponderance (1662 patients, 48.5%). The obliteration analysis included 2834 patients that met follow-up criteria and contained obliteration data. The weighted mean age was 12.63 years. Pooled cohort analysis found no significant difference in obliteration proportions when comparing GK to LINAC (*P* = 0.7449), with an overall obliteration rate of 63% in patients with at least 1 year follow-up. The sub-cohort analysis of all patients treated with SRS revealed that presentation with AVM hemorrhage was associated with increased obliteration (CE: RR = 1.22 [95%CI = 1.09–1.35; RE: RR = 1.22, 95%CI = 10.6–1.40; prediction interval = 1.07–1.38) with low heterogeneity (*I*^2^ = 17.1%, *τ*^2^ < 0.0001, *p* = 0.2902). Smaller SM grade was not statistically associated with increased obliteration (CE: RR = 1.25 [95%CI = 0.87–1.81]; RE: RR = 1.84 [95%CI = 0.97–3.50]; prediction interval = 0.38–8.86) and moderate levels of heterogeneity were detected (*I*^2^ = 45.2%, *τ*^2^ = 0.2668, *p* = 0.1042). Procedure prior to SRS also had higher obliteration rates than no prior procedure (CE: RR = 0.77 [95%CI = 0.61–0.86]; RE: RR = 0.71 [95%CI = 0.54–0.92]; prediction interval = 0.36–1.39) with low to moderate heterogeneity (*I*^2^ = 27.6%, *τ*^2^ = 0.0.0264, *p* = 0.2466).

**Conclusion:**

SRS is a safe and effective treatment for pediatric AVMs. This study suggests that there are no differences in obliteration between GK and LINAC, with increased obliteration in patients with hemorrhage at presentation and procedures prior to SRS treatment. Further multicenter, prospective studies are necessary to dictate future treatment decisions.

**Supplementary Information:**

The online version contains supplementary material available at 10.1007/s00381-025-06835-z.

## Introduction

Pediatricarteriovenous malformations (AVM) are rare occurrences that only compose 3% of all AVMs with overall annual risk of hemorrhage between 2 and 4% [1, 2]. AVMs are characterized by abnormal vascular connections that bypass capillaries creating arteriovenous shunting through tortuous vasculature leading to spontaneous rupture. Treatment approaches to the vascular anomaly in pediatric patients remain controversial, with options including microsurgical resection, endovascular embolization, stereotactic radiosurgery, and/or emergency surgery in the cases of rupture [3]. Treatment decision remains nuanced, determined by acuity of presentation, AVM characteristics, and prior treatment that rely on clinical judgement and acumen. The risk of radiation exposure in younger patients adds to the complexity of pediatric AVM treatment. A 20–50% risk of failed obliteration at 3-to-5-year follow-up reported in SRS treatment of AVMs implicates the importance of proper guidelines to prevent unnecessary radiation that can lead to tumors and other complications, as well as hemorrhage of the residual AVM [4, 5].

The treatment plan of pediatric AVMs requires a combination of clinical acumen with published guidelines to determine the course of treatment due to the dearth of cases. Optimal management remains controversial, with the goal of angiographic obliteration accomplishment highly dependent on complexity of the AVM. While microsurgical resection is the gold standard for pediatric AVM treatment, there remains a paucity of detailed, extensive standardized guidelines for SRS intervention other than its recognition as a safe, definitive treatment [6]. Currently, all surgically accessible AVMs are recommended to undergo microsurgical treatment with embolization and SRS utilized as adjunctive treatment. Multidisciplinary approaches with pediatric neurosurgeons, endovascular neurosurgeons or interventional radiologists, and radiation oncologists is recommended if microsurgical access is unavailable or in the case of high complexity malformations. SRS modalities include Gamma Knife (GK) and Linear Accelerator (LINAC), with both seen as safe treatment options and utility dependent on the type of machine available to the treatment center. To understand the role of SRS therapy in the treatment of pediatric AVMS, we conducted a systematic review of the literature and subsequent meta-analyses to uncover the role of the type of SRS has on obliteration rates as well as sub-cohort analyses on the effect of prior hemorrhage, Spetzler-Martin (SM) [7] grade, and prior procedures on obliteration.

## Methods

### Search criteria

**Fig. 1:**
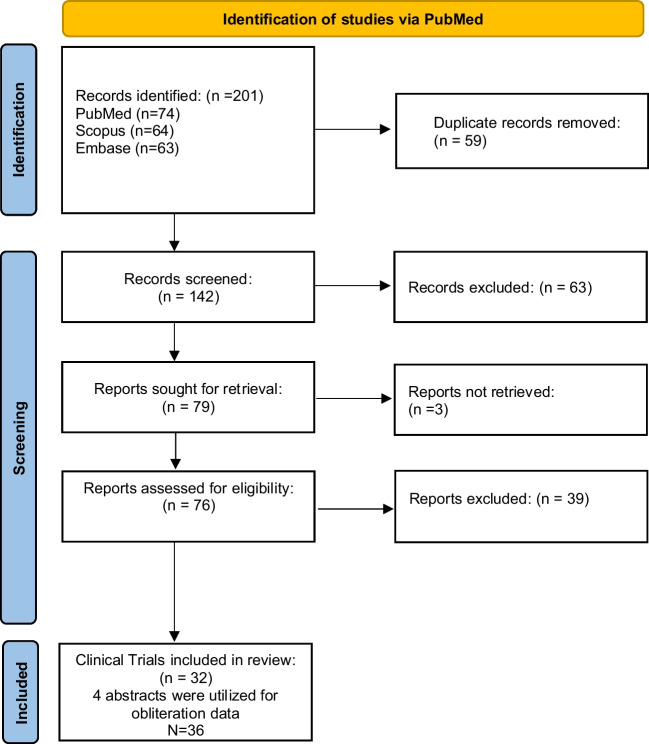
PRISMA diagram illustrating the screening methods, criteria, and literature search to collect pediatric SRS AVM articles

We performed a systematic review of the literature in PubMed, SCOPUS, and Embase to using Title/Abstract and key words to include primary research articles of pediatric patients who underwent SRS treatment of AVMs (Fig. 1). The search terms yielded 201 total articles, with 74 articles from PubMed, 64 from SCOPUS, and 63 from Embase utilizing the following search terms:

- PubMed: (“Pediatric”[tiab] OR “Child”[tiab] OR “Adolescent”[tiab] OR “Infant”[tiab]) AND (“Arteriovenous Malformation” [tiab] OR.
- “Cerebral Arteriovenous malformation” [tiab]) AND (“Radiosurgery” [tiab] OR stereotactic radiosurgery” [tiab] OR “gamma knife” [tiab] OR “LINAC” [tiab] OR “linear accelerator” [tiab] OR “cyber knife” [tiab]).
- SCOPUS: (TITLE (Pediatric) OR TITLE (Child) OR TITLE (Adolescent) OR TITLE (Infant)) AND (TITLE (“Arteriovenous Malformation”) OR TITLE (“Cerebral Arteriovenous malformation”)) AND (TITLE (Radiosurgery) OR TITLE (“Stereotactic Radiosurgery”) OR TITLE- (“Gamma Knife”) OR TITLE-ABS-KEY (LINAC) OR TITLE-ABS-KEY (“Linear Accelerator”) OR TITLE-ABS-KEY (“Cyber Knife”)).
- Embase: (‘pediatric:ab,ti OR child:ab,ti OR adolescent:ab,ti OR infant:ab,ti) AND (‘arteriovenous malformation’:ab,ti OR ‘cerebral arteriovenous malformation’:ab,ti) AND (radiosurgery:ab,ti OR ‘stereotactic radiosurgery’:ab,ti OR ‘gamma knife’:ab,ti OR linac:ab,ti OR ‘linear accelerator’:ab,ti OR ‘cyber knife’:ab,ti).

### Inclusion and exclusion criteria

The 201 articles were uploaded into Covidence screening software, which removed 59 duplicates. The remaining 142 articles’ title and abstracts were screened independently by authors G.T and R.E.E. according to PRISMA guidelines for the following exclusion criteria: (1) animal subjects; (2) records published before 1970; (3) patients 18 or older; (4) or any secondary research articles such as literature reviews or meta-analyses. Articles that contained both pediatric patients and adult patients, but reported the data separately for each were included. A total of 63 articles were excluded. Then full text, English PDFs were sought for retrieval. Three articles were not retrievable. There were 4 articles in non-English texts that provided an English abstract with obliteration data that was included into the obliteration analysis. A second round of independent, full-text screening was performed on the remaining 76 articles using the following inclusion criteria: (1) human subjects younger than 18; (2) records published after 1970; (3) sample size greater than or equal to 10; (4) primary research articles; (5) obliteration rates obtainable; (6) English, full-text pdf obtainable or obliteration data obtainable from English abstract. After screening, we obtained 32 full-text articles and 4 abstracts with sufficient data to analyze SRS treatment of pediatric AVMs. Repeat SRS exposure was also excluded from the meta-analysis of obliteration rates for statistical integrity but was included in the tables. This review was not registered in a database.

### Data collection

Article title, author, number of patients, sex, age, lesion volume, Spetzler-Martin (SM) grade, RS-Based AVM Score, pre-SRS hemorrhage, prior embolization or surgery of AVM, type of SRS, prescribed and marginal dose, follow-up, obliteration rates, presence of aneurysm, AVM diameter, location, radiation-induced changes (RIC), post-SRS hemorrhage, other complications, and length of stay for emergency presentation. Data was independently collected by authors G.T. and R.E.E. according to PRISMA guidelines. Obliteration was confirmed by DSA, MRI, or both.

### Quality assessment

Quality assessment was performed by two independent reviewers using the methodological index for non-randomized studies (MINORS) scale to assess the quality and risk of bias of each article (Table 1). Scores were assigned 0–2 based on 12 criteria, with 16 for non-comparative studies and 24 as the maximum score, respectively. A higher score indicates a lower risk for bias [8].

**Table 1:** Quality assessment using methodological index for non-randomized studies (MINORS) criteria

	Non-comparative study criteria								Comparative Study Criteria				
Article	Clearly stated aim	Inclusion of consecutive patients	Prospective collection of data	Endpoints appropriate to the aim of the study	Unbiased assessment of study endpoint	Follow-up period appropriate to the aim of the study	Loss to follow-up less than 5%	Prospective calculation of the study size	An adequate control group	Contemporary groups	Baseline equivalence of groups	Adequate statistical analyses	**Total Minors Score**
Chen et al. 2018 [9]	2	2	0	2	1	2	2	0	2	2	1	1	**17**
Zabel-du Bois et al. 2006 [10]	2	2	0	2	1	2	2	0	**-**	**-**	**-**	**-**	**11**
Maity et al. 2004 [11]	2	1	0	2	1	2	2	0	**–**	**-**	**-**	**-**	**10**
Reyns et al. 2007 [12]	2	2	0	2	1	2	2	0	**-**	**-**	**-**	**-**	**11**
Nicolato et al. 2015 [13]	2	2	0	2	1	2	2	0	**-**	**-**	**-**	**-**	**11**
Tanaka et al. 1997 [14]	2	1	0	2	1	2	0	0	2	2	1	1	**14**
Potts et al. 2014 [15]	2	1	0	2	2	2	2	0	**-**	**-**	**–**	**-**	**11**
Zeiler et al. 2015 [16]	2	1	0	2	1	2	1	0	**-**	**-**	**-**	**-**	**9**
Patibandla et al. 2017 [17]	2	1	0	2	1	2	2	0	**-**	**-**	**-**	**-**	**10**
Sheth et al. 2014 [18]	2	1	0	2	1	2	2	0	**-**	**-**	**-**	**-**	**10**
Park et al. 2017 [19]	2	2	0	2	1	2	1	0	**-**	**-**	**-**	**-**	**10**
Hanakita et al. 2015 [20]	2	1	0	2	1	2	1	0	**-**	**-**	**-**	**-**	**9**
Nicolato et al. 2005 [21]	2	2	0	2	1	2	1	0	**-**	**-**	**-**	**-**	**10**
Shuto et al. 2008 [22]	**-**	**-**	**-**	**-**	**-**	**-**	**-**	**-**	**-**	**-**	**-**	**-**	**0**
Tamura et al. 2012 [23]	2	1	0	2	1	2	2	0	**-**	**-**	**-**	**-**	**10**
Blamek et al. 2013 [24]	2	2	0	2	1	2	2	0	**-**	**-**	**-**	**-**	**11**
Rajshekhar et al. 2016 [25]	2	1	0	2	1	2	1	0	**-**	**-**	**-**	**-**	**9**
Yeon et al. 2011 [26]	2	1	0	2	1	2	1	0	**-**	**-**	**-**	**-**	**9**
Glazener et al. 2020 [27]	2	2	0	2	1	2	1	0	**-**	**-**	**-**	**-**	**10**
Hasagawa et al. 2019 part 1 [28]	2	1	0	2	1	2	1	0	**-**	**-**	**-**	**-**	**9**
Starke et al. 2017 [29]	2	2	0	2	1	2	1	0	**-**	**-**	**-**	**-**	**10**
Chen et al. 2020 [30]	2	1	0	2	1	2	0	0	**-**	**-**	**-**	**-**	**8**
Hasagawa et al. 2019 part 2 [31]	2	1	0	2	1	2	1	0	**-**	**-**	**-**	**-**	**9**
Monteith et al. 2011 [32]	**-**	**-**	**-**	**-**	**-**	**-**	**-**	**-**	**-**	**-**	**-**	**-**	**0**
Kano et al. 2012 [33]	2	1	0	2	1	2	2	0	**-**	**-**	**-**	**-**	**10**
Buis et al. 2008 [34]	2	2	0	2	1	2	2	0	**-**	**-**	**-**	**-**	**11**
Nataf et al. 2001 [35]	**-**	**-**	**-**	**-**	**-**	**-**	**-**	**-**	**-**	**-**	**-**	**-**	**0**
Ding et al. 2015 [36]	2	1	0	2	1	2	2	0	**-**	**-**	**-**	**-**	**10**
Burke et al. 2020 [37]	2	1	0	2	1	2	2	0	**-**	**-**	**-**	**-**	**10**
Chen, Lee et al. 2020 [38]	2	1	0	2	1	2	2	0	**-**	**-**	**-**	**-**	**10**
Nataf et al. 2003 [39]	2	1	0	2	1	2	1	0	**-**	**-**	**-**	**-**	**9**
Lee et al. 2021 [40]	**-**	**-**	**-**	**-**	**-**	**-**	**-**	**-**	**-**	**-**	**-**	**-**	**0**
Börcek et al. 2014 [41]	2	1	0	2	1	2	2	0	**-**	**-**	**-**	**-**	**10**
Chen et al. 2019 [42]	2	1	0	2	1	2	2	0	**-**	**-**	**-**	**-**	**10**
Burke et al. 2021 [43]	2	1	0	2	1	2	2	0	**-**	**-**	**-**	**-**	**10**
Garcia et al. 2024 [44]	2	1	0	2	1	2	2	0	**-**	**-**	**-**	**-**	**10**

### Analysis

Results from the included studies were synthesized through meta-analysis to pool the effect sizes. The risk ratios were pooled using a restricted maximum-likelihood estimation random-effects model with inverse variance weighting and Hartung Knapp adjustment to account for variation between studies. Trials that were missing follow-up information were not included into the analysis.

Study variance between studies was assessed with Cochranes *χ*^2^ and magnitude of any observed heterogeneity between studies was assessed using the *I*^2^ statistic. Publication bias was analyzed via the Egger test and visually assessed utilizing funnel pots. All statistical analysis was performed using R statistical software (version 4.3.1, R Foundation for Statistical Computing, Vienna, Austria). We utilized the *meta* package in our analysis.

## Results

### Aggregate demographics and characteristics

A total of 3,25 unique patients were included in the study, 2834 patients with proper obliteration data and follow-up met criteria to determine obliteration rates (Table 2). The 3425 patients were utilized to calculate qualitative percentages of study characteristics (Tables 2, 3, and 4). Some manuscripts reused previously published cohorts with differing variables reported. These manuscripts were included, but aggregate data was only counted once [28, 30, 31, 38, 42, 43]. There is a slight male preponderance 1662 patients (48.5%) compared to female patients (1481, 43.2%) with some sex data missing (282). Weighted mean age of the cohort is 12.67 years. The weighted mean lesion volume is 4.62 cm^3^. The cohort has 2536 AVMs with a SM grade of 1–3 (74.0%) and 474 SM graded 4–5 (13.8%) with 415 SM grades missing (12.1%). Location of AVM was not well reported and thus not included in the analysis. The most common SRS modality is GK (2511) while fewer patients were treated with LINAC (323 patients) (Table 2). Patients were not included in this count if at least 12-month follow-up was not reported. One study utilized proton beam therapy [40]. There were 1785 patients (63%) with obliterated AVMs with at least 12-month follow-up (Table 2).

**Table 2:** Aggregate demographic data including number of patients, age, sex, lesion volume, SM grade, SRS modality, complications, and obliterations

Variable	Value
**Total # of patients**	**3425**
**Sex**	
Male	1662 (48.5%)
Female	1481 (43.2%)
**Weighted mean age (years)**	**12.63**
**Weighted mean lesion volume (cm**^**3**^**)**	**4.21**
**SM grade**	
SM grade I–III	2536 (74.0%)
SM grade IV–V	474 (13.8%)
Not reported	415 (12.1%)
**SRS modality**	
GK	2511
LINAC	323
Proton beam	24
**Complication**	**526 (15.3%)**
Radiation-induced changes	172
Post-SRS hemorrhage	158
Nausea/vomiting	3
Headache	34
Necrosis	11
Seizure	28
Visual defects	2
Cyst	18
Edema	19
Tumor	6
Other	66
Death	9
**Obliterations**	**1785 (63%)**

*SM*, Spetzler-Martin; *SRS*, stereotactic radiosurgery

**Table 3:** Individualized demographic and characteristics

Citation	Included patients	Male	Female	Age median (years)	Age range (years)	Age means (years)	Age SD (years)	Median lesion volume (cm^3^)	Mean lesion volume (cm^3^)	SD lesion volume (cm^3^)	SM 1–3	SM 4–5
Chen et al. 2018 [9]	346	186	160	**-**	**-**	12.4	3.6	**-**	3.5	3.5	307	39
Zabel-du Bois et al. 2006 [10]	22	11	11	11.8	4.4–16.4	**-**	**-**	4.2	**-**	**-**	20	2
Maity et al. 2004 [11]	17	8	9	12	5.0–18	**-**	**-**	**-**	**-**	**-**	**-**	**-**
Reyns et al. 2007 [12]	100	56	44	12	2.0–16	**-**	**-**	**-**	2.8	**-**	68	5
Nicolato et al. 2005 [21]	100	46	54	**-**	3.0–18	12.8	**-**	**-**	2.8		92	8
Tanaka et al. 1997 [14]	23	**-**	**-**	**-**	2.0–15	11.5	**-**	**-**	4.2	**-**	23	1
Potts et al. 2014 [15]	80	49	31	**-**	**-**	12.7	3.8	**-**	8.4	12.2	55	20
Zeiler et al. 2015 [16]	15	6	9	15	7–18	14.2		2.4	3.1	**-**	9	0
Patibandla et al. 2017 [17]	28	15	13	**-**	**-**	12.1	3.7	**-**	5.9	4.4	0	28
Sheth et al. 2014 [18]	42	24	18	**-**	**-**	12	4	**-**		**-**	24	18
Park et al. 2017 [19]	68	35	33	**-**	4.0–18	13.3	**-**	**-**	2.1	**-**		
Hanakita et al. 2015 [20]	116	52	64	14	4.0–18	**-**	**-**	1.8	**-**	**-**	100	12
Nicolato et al. 2005 [21]	63	27	36	**-**	5.0–16	11.7	**-**	**-**	3.8		35	5
Shuto et al. 2008 [22]	43	15	28	**-**	**-**	11.7	**-**	**-**	4.5		38	5
Tamura et al. 2012 [23]	22	14	8	9.5	4.0–14	**-**	**-**	1.2	2.7		14	6
Blamek et al. 2013 [24]	10	4	6	**-**	8.0–18	15.4	**-**	**-**	13.2		8	2
+ Rajshekhar et al. 2016 [25]	82	36	33	14	7.0–18	**-**	**-**	**-**	8.5	8.7	59	10
Yeon et al. 2011 [26]	39	21	18	**-**	3.0–17	12.2	**-**	**-**	1.5	**-**	29	10
Glazener et al. 2020 [27]	34	19	15	14.4	5.5–17.9	**-**	**-**	2.91	**-**	**-**	28	6
**Hasegawa et al. 2019 [28]	189	112	77	11	**-**	**-**	**-**	2.2	**-**	**-**	168	21
Starke et al. 2017 [29]	357	194	163	15.1	**-**	12.6	**-**	**-**	3.5	3.3	314	45
*Chen et al. 2020 [30]	539	284	255	13.3	**-**	12.8	**-**	3	5.9	**-**	456	83
**Hasegawa et al. 2019 [31]	189	112	77	11	**-**	**-**	**-**	**-**	**-**	**-**	168	21
Monteith et al. 2011 [32]	186	**-**	**-**	**-**	**-**	**-**	**-**	**-**	3.2	**-**	**-**	**-**
Kano et al. 2012 [33]	135	75	60	12	2.0–17	**-**	**-**	**-**	**-**	**-**	95	40
Buis et al. 2008 [34]	22	15	7	13.8	**-**	13	**-**	1.8	2.9	**-**	20	2
Nataf et al. 2001 [35]	55	**-**	**-**	**-**	**-**	**-**	**-**	**-**	4	**-**	47	8
Garcia et al. 2024 [44]	83	42	41	First SRS 11; second SRS 15	First SRS 3.0–17; second SRS 12–17	**-**	**-**	First SRS: 4.5; second SRS: 1.6	**-**	**-**	**-**	**-**
Ding et al. 2015 [36]	51	27	24	**-**	**-**	12.6	3.6	**-**	3.9		43	8
Burke et al. 2020 [37]	345	182	163	**-**	**-**	12.8	3.1	**-**	4.3	6.6	298	47
Chen et al. 2020 [30]	101	62	39	**-**	**-**	13.5	**-**	**-**	9	11.9	84	17
Nataf et al. 2003 [39]	49	21	28	12	**-**	**-**	-	3.5	3	**-**	44	5
Lee et al. 2021 [40]	24	**-**	**-**	14	**-**	**-**	**-**	8.4	**-**	**-**	19	5
Börcek et al. 2014 [41]	58	24	34	12	**-**	12.41	**-**	3.5	4.99	4.53	42	16
*Chen et al. 2019 [42]	539	284	255	**-**	**-**	**-**	**-**	**-**	**-**	**-**	456	83
*Burke et al. 2021 [43]	539	284	255	**-**	**-**	**-**	**-**	**-**	**-**	**-**	456	83

*SD*, standard deviation; *SM*, Spetzler-Martin. */**Same cohort with some different sub-cohort data reported

**Table 4:** Treatment characteristics

Citation	Hemorrhage/rupture prior to radiosurgery	Prior embolization	Prior surgery	Prior surgery and embolization	Type of SRS	Median prescription dose (Gy)	Mean prescription dose (Gy)	Range prescription dose (Gy)	Marginal dose median (Gy)	Marginal dose mean (Gy)	Marginal dose range (Gy)	Mean maximal dose (Gy)	Median maximal dose (Gy)	Range maximal dose (Gy)
Chen et al. 2018 [9]	234	71	21	**-**	GK	**-**	**-**	**-**	**-**	21	**-**	39.5	**-**	**-**
Zabel-du Bois et al. 2006 [10]	13	5	1	**-**	LINAC	**-**	**-**	**-**	18	**-**	15–20	**-**	**-**	**-**
Maity et al. 2004 [11]	13	5	3	**-**	LINAC	**-**	17.58	16–18	**-**	**-**	**-**	**-**	**-**	**-**
Reyns et al. 2007 [12]	69	38	6	2	LINAC	**-**	**-**	**-**	**-**	23	15–25	**-**	**-**	**-**
Nicolato et al. 2005 [21]	70	32	6	3	GK	**-**	**-**	**-**	**-**	20.2	9–26.4	37.8	**-**	18–50
Tanaka et al. 1997 [14]	21	0	5	**-**	GK	**-**	**-**	**-**	**-**	20.5	**-**	36.8	**-**	**-**
Potts et al. 2014 [15]	45	10	8	**-**	GK	17.5	17.1	12.0–20	**-**	**-**	**-**	**-**	**-**	**-**
Zeiler et al. 2015 [16]		2	1	**-**	GK	**-**	**-**	**-**	20	19.5	16–22	39	40	32–44
Patibandla et al. 2017 [17]	18	3	4	**-**	GK	**-**	**-**	**-**	**-**	19.4	**-**	36.9	**-**	**-**
Sheth et al. 2014 [18]	16	9	2	**-**	GK	17	**-**	12.0–20	**-**	**-**	**-**	**-**	**-**	**-**
Park et al. 2017 [19]	53	3	14	**-**	GK	**-**	**-**	**-**	**-**	22.9	18–26	37.6	**-**	26.6–50
Hanakita et al. 2015 [20]	88	13	34	**-**	GK	**-**	**-**	**-**	20	**-**	12.5–25	**-**	40	25–60
Nicolato et al. 2005 [21]	50	17	2	1	GK	**-**	21.6	16–26	**-**	**-**	**-**	39.7		24–50
Shuto et al. 2008 [22]	**-**	8	5	**-**	GK	**-**	**-**	**-**	**-**	19.9	12–25	**-**	**-**	**-**
Tamura et al. 2012 [23]	17	2	1	1	GK	**-**	**-**	**-**	22	22.6	20–25	48.8	44	36.3–50
Blamek et al. 2013 [24]	6	**-**	**-**	**-**	LINAC	**-**	**-**	**-**	**-**	**-**	16–24	**-**	**-**	**-**
Rajshekhar et al. 2016 [25]	**-**	9	**-**	**-**	LINAC	**-**	**-**	**-**	15	**-**	9–20	**-**	**-**	**-**
Yeon et al. 2011 [26]	25	**-**	**-**	**-**	GK	**-**	**-**	**-**	20	21.2	13–30	**-**	**-**	**-**
Glazener et al. 2020 [27]	22	5	4	**-**	LINAC	16.8	**-**	14–20	**-**	**-**	**-**	**-**	**-**	**-**
**Hasegawa et al. 2019 [28]	157	27	43	**-**	GK	**-**	**-**	**-**	20	**-**	11–30.6	**-**	37	22–55
Starke et al. 2017 [29]	245	78	23	**-**	GK	**-**	**-**	**-**	**-**	21	**-**	39.5	**-**	**-**
*Chen et al. 2020 [30]	386	91	33	**-**	GK	**-**	**-**	**-**	20	20.2	**-**	36.7	36	**-**
**Hasegawa et al. 2019 [31]	157	27	43	**-**	GK	**-**	**-**	**-**	20	**-**	11–30.6	**-**	37	22–55
Monteith et al. 2011 [32]	133	**-**	**-**	**-**	GK	**-**	21.9	**-**	**-**	**-**	**-**	**-**	**-**	**-**
Kano et al. 2012 [33]	87	25	**-**	**-**	GK	**-**	**-**	**-**	20	**-**	15–25	**-**	40	30–50
Buis et al. 2008 [34]	19	**-**	**-**	**-**	LINAC	19	18.8	**-**	**-**	**-**	**-**	**-**	**-**	**-**
Nataf et al. 2001 [35]	**-**	**-**	**-**	**-**	**-**	**-**	**-**	**-**	**-**	**-**	**-**	**-**	**-**	**-**
Garcia et al. 2024 [44]	First SRS:41, Second SRS 42	15	6	**-**	GK	First SRS: 19; second SRS: 19	**-**	First SRS:7.5–36 Second SRS: 14–33	**-**	**-**	**-**	**-**	**-**	**-**
Ding et al. 2015 [36]	0	14	3	**-**	GK	**-**	**-**	**-**	21.5	21.3	14–27	40	40	27–50
Burke et al. 2020 [37]	**-**	39	17	**-**	GK	**-**	**-**	**-**		20.8	**-**	37.5	**-**	**-**
Chen et al. 2020 [30]	**-**	**-**	**-**	**-**	GK	**-**	**-**	**-**	**-**	19.2	**-**	**-**	**-**	**-**
Nataf et al. 2003 [39]	**-**	14	9	**-**	LINAC	**-**	**-**	**-**	**-**	**-**	**-**	35.3	35.7	28.6–42.7
Lee et al. 2021 [40]	**-**	**-**	**-**	**-**	PB	**-**	**-**	**-**	**-**	**-**	**-**	**-**	**-**	**-**
Börcek et al. 2014 [41]	24	11	15	**-**	GK	22	20.77	15–24	**-**	**-**	**-**	42.09	44	31.4–48.5
*Chen et al. 2019 [42]	386	91	33	**-**	GK	**-**	**-**	**-**	**-**	**-**	**-**	**-**	**-**	**-**
*Burke et al. 2021 [43]	386	91	33	**-**	GK	**-**	**-**	**-**	**-**	**-**	**-**	**-**	**-**	**-**

*GK*, Gamma Knife; *LINAC*, linear accelerator; *PB*, proton beam; *SRS*, stereotactic radiosurgery. */**Same cohort with some different sub-cohort data reported

Complications occurred in 526 patients (15.3%). Almost one-third of these complications were radiation-induced changes (172 patients) which varied from mild and transient to severe. However, the severity of these changes was not homogenously reported. Post-SRS hemorrhage comprised another third of the complications (158 patients), but there was insufficient information reported on severity of hemorrhage. Other complications noted were mild, including nausea and vomiting (3 patients) and headache (34 patients). More severe complications included seizure (28 patients), radiation necrosis (11 patients), visual defects (2 patients), cysts (18 patients), edema (19 patients), tumor (6 patients), and death (9 patients) (Table 2). Of the tumors mentioned, 4 were meningiomas observed more than 10 years after SRS treatment while 1 glioblastoma multiforme and 1 anaplastic oligodendroglioma were observed at around 7 years post-SRS.

### Individualized demographics, AVM characteristics, and SRS dosage

Most studies report around 100 patients, with some studies including large multicenter cohorts and others including smaller case series (Table 3). One of the articles report patients undergoing their second SRS treatment [44], while the rest report patients treated with SRS for the first time. The median age ranges from 9.5 to 15.1 years. The range of the entire cohort is 2 to 17.9 years. The median lesion volumes range from 1.2 to 8.4 cm^3^ while the mean lesion volume range from 1.5 to 9 cm^3^. We also report standard deviations of lesion volumes (Table 3).

One-thousand forty-two patients presented with hemorrhage or rupture prior to SRS (Table 4). Prior treatment is reported among 819 patients, 546 of those patients with prior embolization, 266 prior surgeries, and 7 both surgery and embolization. Eighty-three patients previously received SRS but were not included in the sub-cohort analysis in Fig. 5. The range of median prescription dosages is 16.8 to 22 Gy and mean prescription dose of 17.1 to 21.9 Gy. The median marginal dose ranges from 15 to 22 Gy and mean range from 19.2 to 23 Gy. The maximal SRS dose median ranges from 35.7 to 40 Gy and mean maximal dose ranges from 35.3 to 42.09 Gy (Table 4).

### GK vs LINAC

**Fig. 2:**
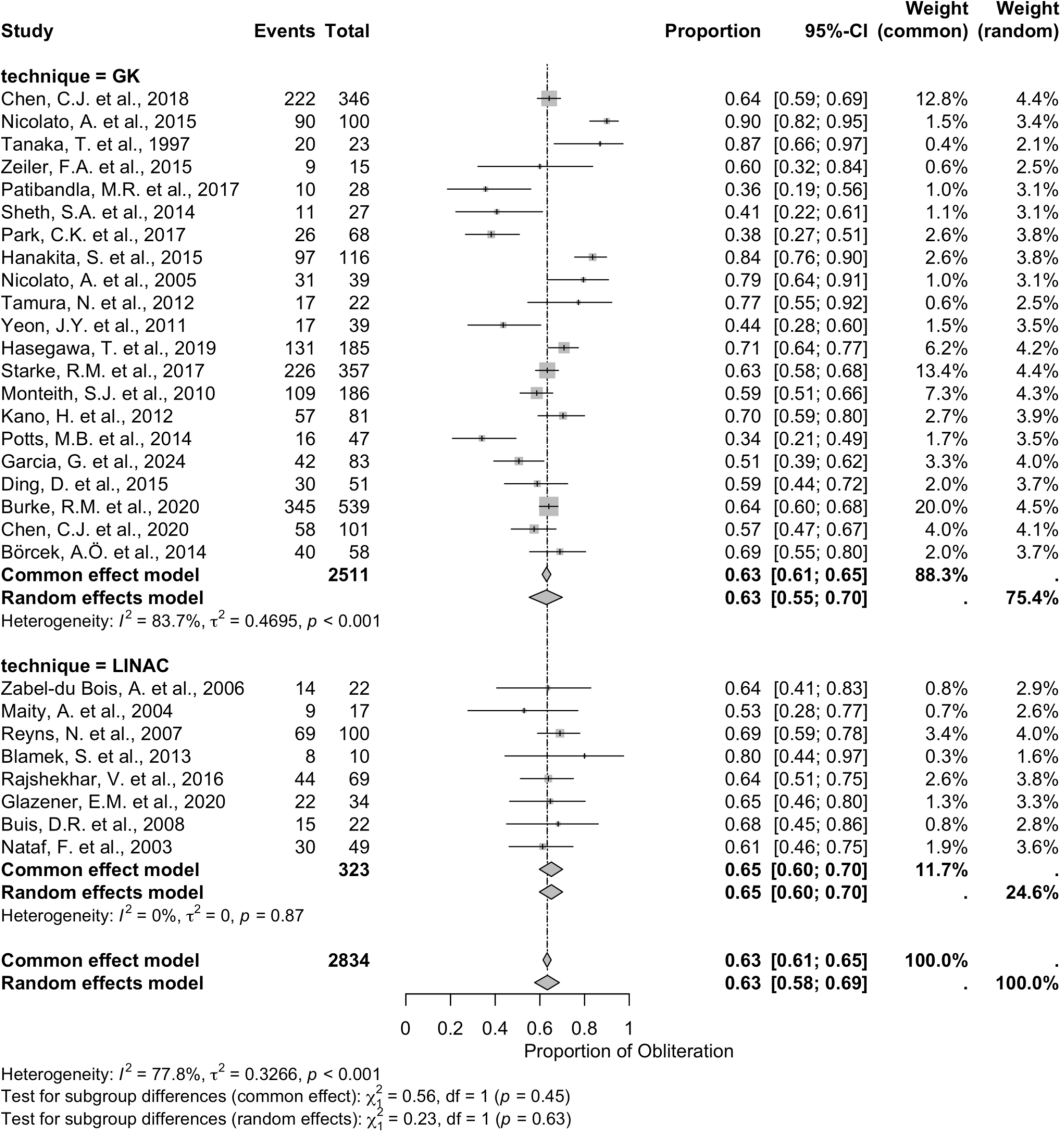
Forest plot illustrating risk ratio for obliteration in patients treated with GK or LINAC. QM (df = 1) = 0.1059, *p* = 0.7449. *CI*, confidence interval; *GK*, Gamma Knife; *LINAC*, linear accelerator

All studies that utilized GK or LINAC reported individualized obliteration rates that show no significant difference (QM (df = 1) = 0.1059, *p* = 0.7449) in obliteration prevalence between GK (proportion; common effects (CE) = 0.63 [95% CI = 0.61–0.65], random effects (RE) = 0.63 [95% CI = 0.55–0.70]) and LINAC (Proportion; CE = 0.65 [95% CI = 0.60–0.70], RE = 0.65 [95% CI = 0.60–0.70]). Total number of SRS treatments does not match total number of patients due to lack of follow-up. Overall obliteration prevalence provided similar rates (proportion; CE = 0.63 [95% CI = 0.61–0.65], RE = 0.63 [95% CI = 0.58–0.69]. Significant heterogeneity was found among GK studies (*I*^2^ = 83.7%, *τ*^2^ = 0.4695, *p* < 0.001) while prevalence of the LINAC studies was mostly due to chance, not heterogeneity (*I*^2^ = 0%, *τ*^2^ = 0, *p* = 0.87). In general, studies demonstrated significant heterogeneity, likely due to the high heterogeneity of GK models (*I*^2^ = 77.8%, *τ*^2^ = 0.3266, *p* < 0.001). There were no significant differences between subgroups (CE: *χ*^2^ = 0.56, df = 1, *p* = 0.45; RE: *χ*^2^ = 0.23, df = 1, *p* = 0.63) (Fig. 2). This cohort has low risk of publication bias (Supplemental Fig. 1).

### Hemorrhage, SM grade, and prior procedure

**Fig. 3:**
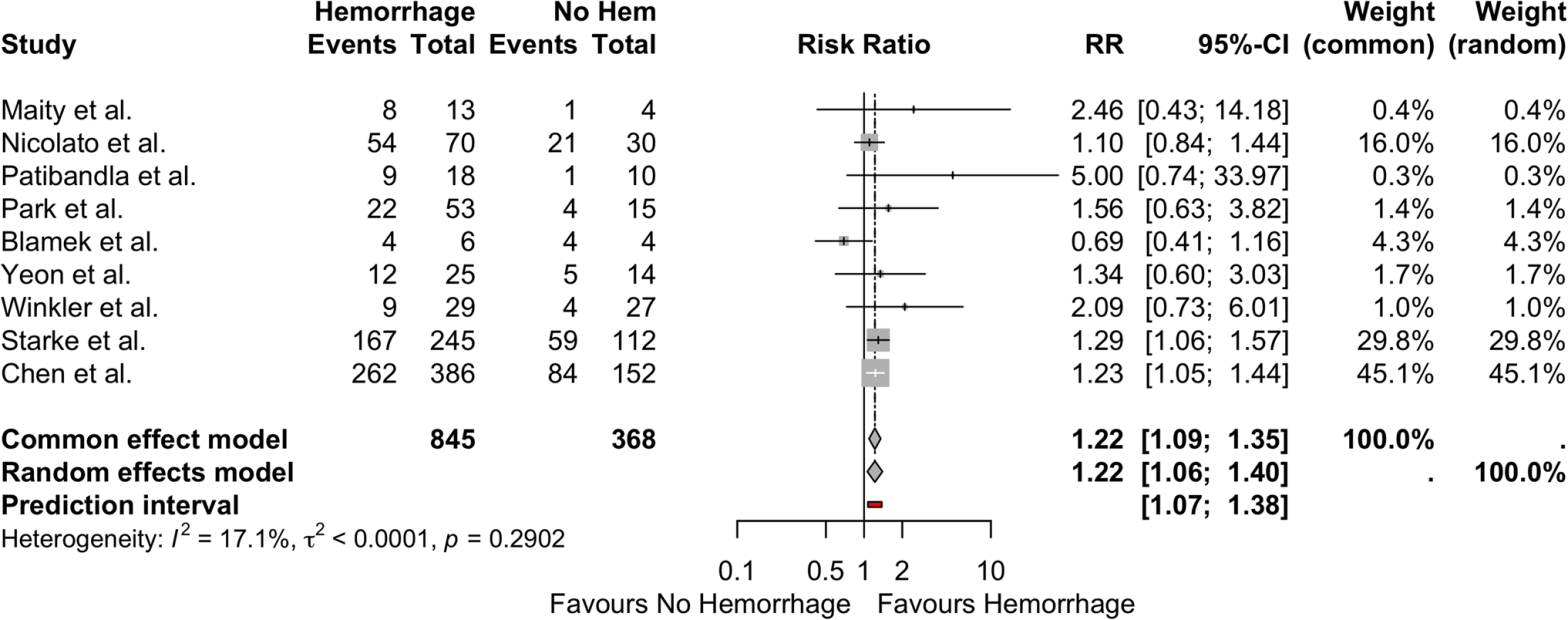
Forest plot illustrating risk ratio for obliteration in patients with hemorrhage prior to SRS. *CI*, confidence interval; *RR*, risk ratio

Nine studies included adequate obliteration data that corresponded with presence or absence of prior hemorrhage. Hemorrhage before SRS has higher obliteration rates than patients with no reported hemorrhage in the common effects model (RR = 1.22 [95%CI = 1.09–1.35]) and random-effects model (RR = 1.22, 95%CI = 10.6–1.40) (prediction interval = 1.07–1.38). This model has significantly low levels of heterogeneity (*I*^2^ = 17.1%, *τ*^2^ < 0.0001, *p* = 0.2902) and publication bias (Figs. 3 and Supplemental Fig. 2).

**Fig. 4:**
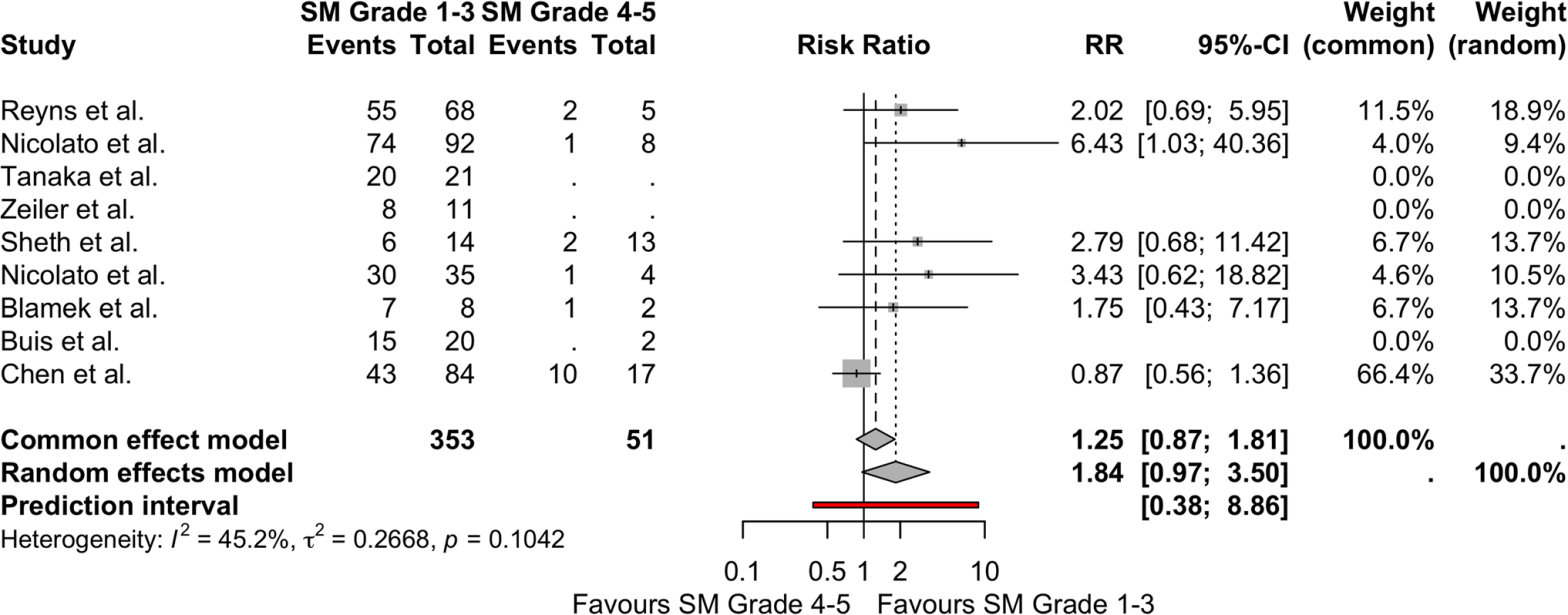
Forest plot illustrating risk ratio for obliteration in patients with SM grade 1–3 compared to SM grade 4–5. *CI*, confidence interval; *RR*, risk ratio; *SM*, Spetzler-Martin

Nine studies included adequate obliteration data that was able to be separated into SM grading for analysis. An SM grade 1–3 has no statistically significant chances of obliteration compared to SM grade 4–5 in the common effects model (RR = 1.25 [95%CI = 0.87–1.81]) or in the random-effects model (RR = 1.84 [95%CI = 0.97–3.50]) (prediction interval = 0.38–8.86). Moderate levels of heterogeneity were detected in these studies (*I*^2^ = 45.2%, *τ*^2^ = 0.2668, *p* = 0.1042) with moderate levels of publication bias. (Figs. 4 and Supplemental Fig. 3).

**Fig. 5:**
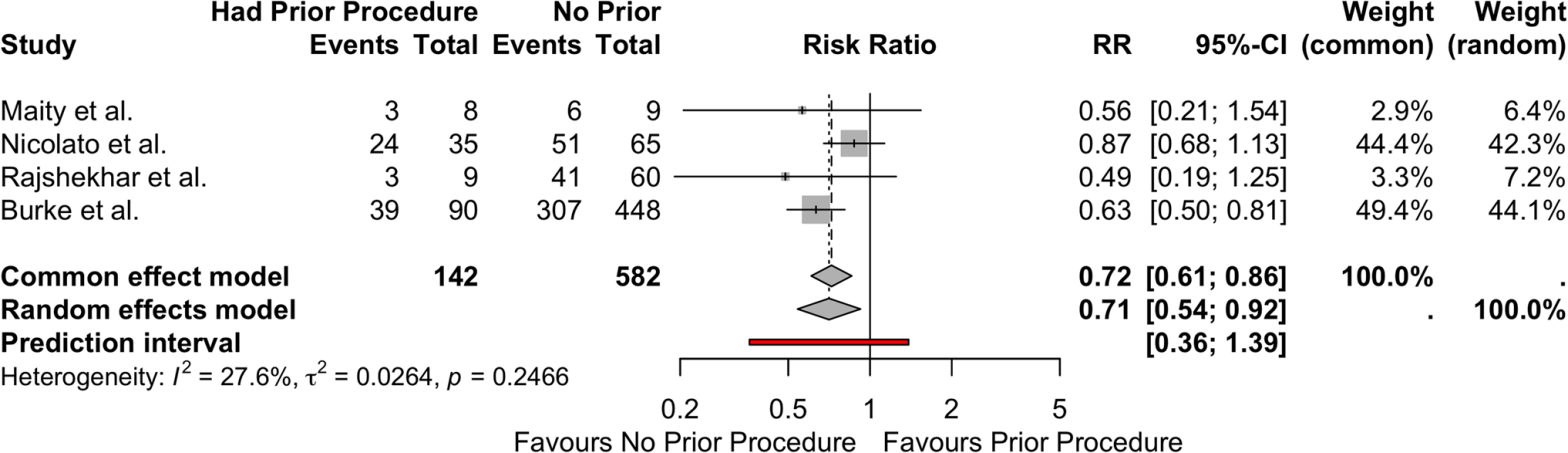
Forest plot illustrating risk ratio for obliteration in patients with prior procedure. *CI*, confidence interval; *RR*, risk ratio

Four studies portrayed enough obliteration data in relation to prior procedures to perform a sub-cohort analysis. Prior procedure, either embolization, surgery, or both, decrease the chance of obliteration due to SRS in both CE model (RR = 0.77 [95%CI = 0.61–0.86]) and RE models (RR = 0.71 [95%CI = 0.54–0.92]) (prediction interval = 0.36–1.39). The studies included in this analysis exhibited low to moderate heterogeneity (*I*^2^ = 27.6%, *τ*^2^ = 0.0.0264, *p* = 0.2466) with possible publication bias (Figs. 4 and 5).

## Discussion

We performed a systematic review and meta-analysis in pediatric AVM to determine the relationship between type of SRS, prior hemorrhage, SM grade, and prior procedure on obliteration rates in pediatric AVMs. While many of the articles were only available to include in the pooled analysis on the SRS modality, a sub-cohort analysis of hemorrhage, SM grade, and prior procedure was possible.

SRS is a safe and effective treatment modality in adult patients, especially for lower SM grade AVMs with obliteration rates. Obliteration rates as high as 80% in SM grades 1 and 2 are possible, with obliteration rates ranging from 50 to 90% in SM grades 3 [45, 46]. The International Stereotactic Radiosurgery Society (ISRS) has developed extensive guidelines in the treatment of AVMs in adult patients, with recent guidelines released for repeat SRS in adult populations [47]. However, there is a paucity of guidelines available for the pediatric treatment of AVMs until a recent report by the ISRS establishing Level 4 evidence for SRS as a definitive treatment option in these patients [6]. The ISRS meta-analysis found an obliteration rate of 80%, relatively higher than our study and other recent meta-analyses obliteration rates of around 60–70% [6, 48, 49]. The ISRS study found no statistically significant differences in obliteration rates among in sub-analyses but did find that the average dose was slightly higher in studies with higher obliteration rates [6]. Treatment with SRS in pediatric patients was previously under scrutiny due to ethical considerations of subjecting the patients to radiation. The low complication rate and no increased rate of malignancy following GK, seen in our study at 0.18%, following the ISRS guidelines suggest SRS as a viable, safe treatment option in pediatric patients. While SRS is considered a safe an effective treatment, multi-faceted approaches with radiation oncology, neurointerventionalists, and pediatric neurosurgeons are still necessary to determine the best approach on a case-by-case basis. More complex AVMs may require all two or more approaches with adjunctive treatment to secure angiographic obliteration.

We found no significant difference in the pooled analysis of GK vs LINAC, with an obliteration rate of around 63–64% in both (Fig. 2). Most centers only have one of the types of SRS, so obliteration rates were determined to show similarities of obliteration rates. While no current literature was found that compares the obliteration rates of GK to LINAC, a difference in the dose response curve was seen with a common plateau above a marginal dose of 20 Gy [50]. Pediatric AVMs are the most common cause of hemorrhage in pediatric patients, with higher risk of hemorrhage than adult patients [48]. Increased risk was found to be significantly associated with smaller size, high SM grade, deep venous drainage, single feeders, deep locations, infratentorial lesions, and diffuse morphologies [51]. Many of these features are also associated with indications for SRS due to characteristics that can increase surgical and endovascular complexity [47]. This might explain the high rate of hemorrhage upon presentation for SRS treatment [48]. We found that hemorrhage was associated with higher obliteration rates (RR = 1.22). This is possibly due to the smaller size of AVM which are associated with increased hemorrhage. This could also be due to the inclusion of SM grade 3 into the smaller SM grades, with other studies only including SM grades 1 and 2. While this inclusion of SM grade 3 AVMs into SM grades 1 and 2 is not ideal, the nature of the analysis required dichotic separation of the variable.

Our analysis found that obliteration rates were highest in patients with no prior procedure, whether surgery or embolization (RR = 0.77, 0.71). These findings suggest that SRS as definitive treatment may provide superior obliteration rates when compared to adjunct treatment if SRS is indicated. This suggestion must be taken cautiously, with future prospective studies with more concrete evidence necessary to guide future treatment plans of pediatric AVMs. Other explanations may be that the patients who underwent prior procedure had more complex AVMs that generally have lower obliteration rates. In contrast, other studies illustrate lower complication and mortality rates in multimodal treatment [49]. While our study did not analyze complications, only obliteration rates, the higher complications could be due to asymptomatic RICs intrinsic to SRS treatment [48]. To discern further details of the treatment of pediatric AVMs with SRS, a multicenter prospective database is necessary to determine more extensive guidelines for SRS indications and dosages to decrease complication rates and increase obliteration rates.

This study is subject to many limitations such as publication bias, inclusion of mainly retrospective studies, inconsistent reporting methods, and human error during data collection. The publication bias in this review was addressed through a funnel plot to determine the risk of bias (Supplemental Figs. 1–3). Conference abstracts were also included in an attempt to address publication bias. There is also bias due to the meta regression utilized in this analysis and necessary caution must be utilized when interpreting the results. The quality of each article was assessed as having moderate to high risk of bias according to the MINORS criteria, likely due to their retrospective nature. This study implemented PRISMA guidelines to lower the risk of bias introduced by human error (Table 1). The inconsistent reporting limited the availability of sub-cohort analysis due to missing data. The missing data highlights the importance of subject level and sub-cohort level granularity in retrospective studies to provide higher levels of evidence in future meta-analyses.

## Conclusion

Pediatric AVMs are rare vascular anomalies with an obliteration rate of around 60–70% when treated with SRS, with no differences between LINAC and GK treatment. An increased chance of obliteration is associated with hemorrhage upon presentation and no prior procedures. Treatment of AVMs with SRS should be considered upon these presentations, with treatment recognized as safe and effective by the ISRS.

## Supplementary Information

Below is the link to the electronic supplementary material.

ESM 1 (DOCX 51.5 MB)

## Data Availability

No datasets were generated or analysed during the current study.
