# Supplementary material for: Stereotactic radiosurgery treatment of pediatric arteriovenous malformations: a PRISMA systematic review and meta-analysis
Source: Childs Nerv Syst. 2025 May 23;41(1):188. doi: 10.1007/s00381-025-06835-z (PMC12102109; doi:10.1007/s00381-025-06835-z)
Supplement: Supplementary file 1 — (DOCX 51.5 MB) [file 381_2025_6835_MOESM1_ESM.docx]

**Supplemental**


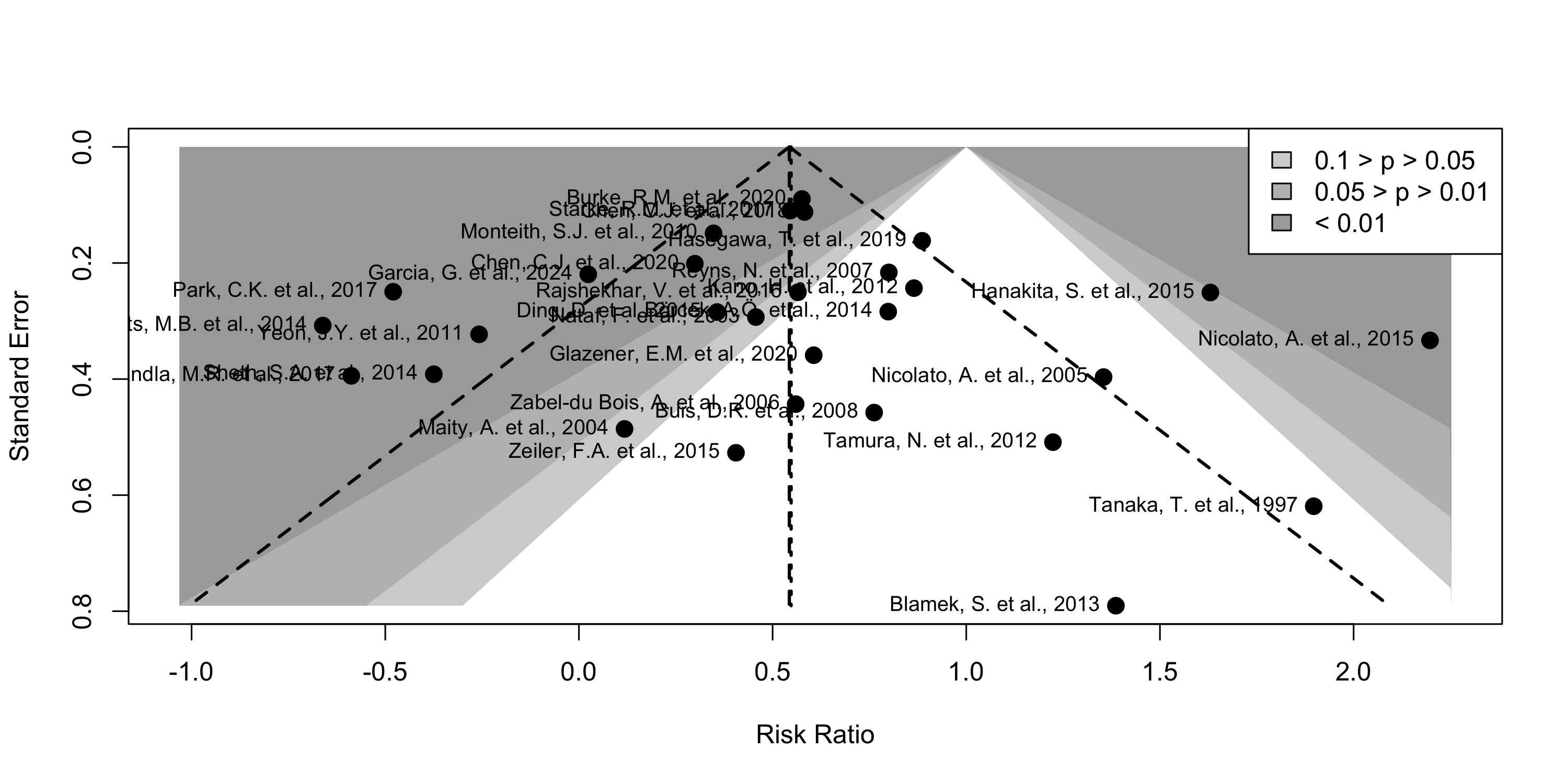


Supplemental Figure 1. Funnel Plot indicating risk of publication bias in analysis ran on GK vs LINAC in Figure 2. Egger’s test not feasible for primary analysis of meta-regression, but symmetry suggests relatively low heterogeneity.


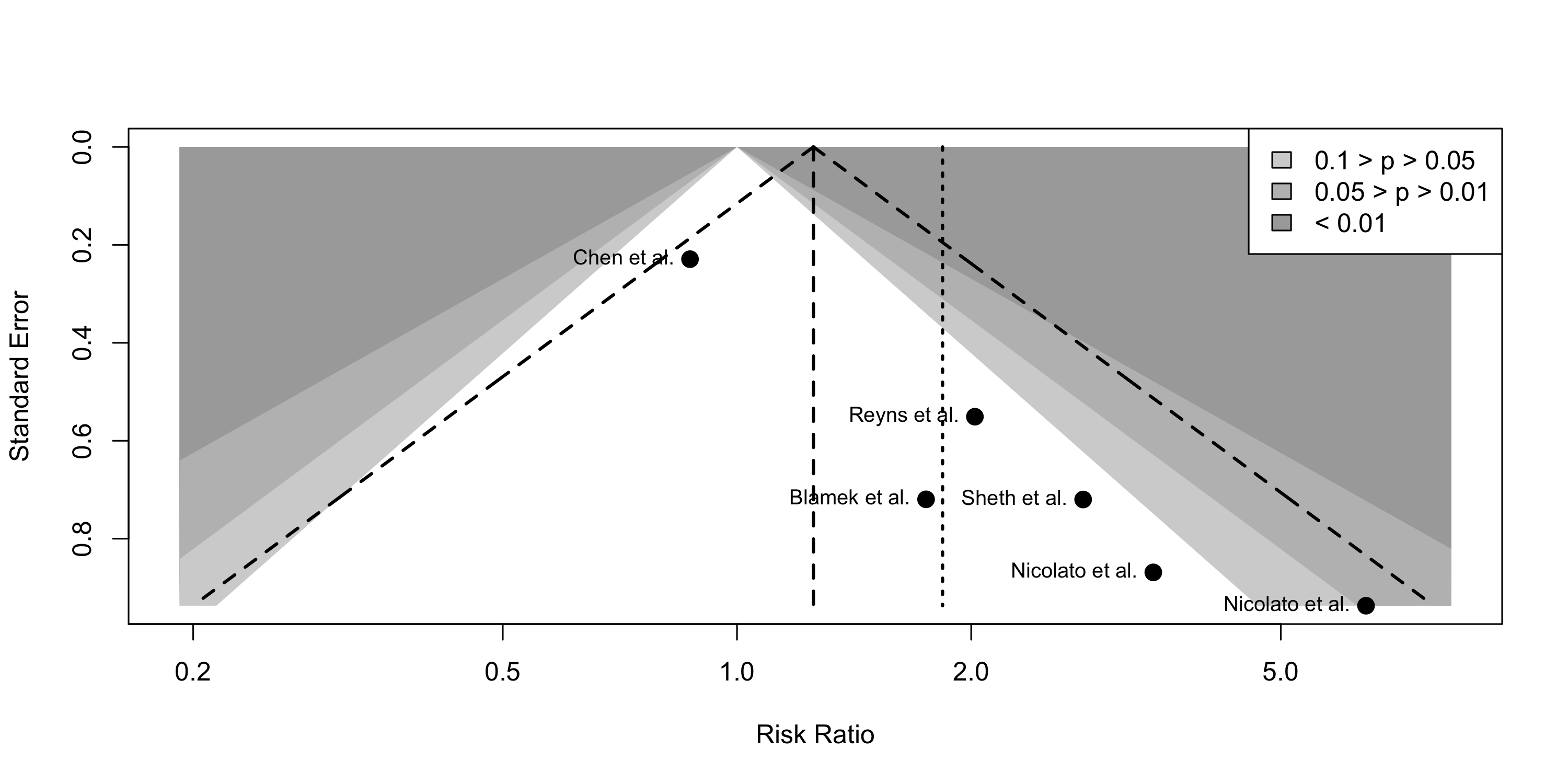


Supplemental Figure 2. Funnel Plot indicating risk of publication bias in hemorrhage analysis in Figure 3. t=0.86, df=7, p=0.4160, bias estimate=0.4985 (SE=0.5767).


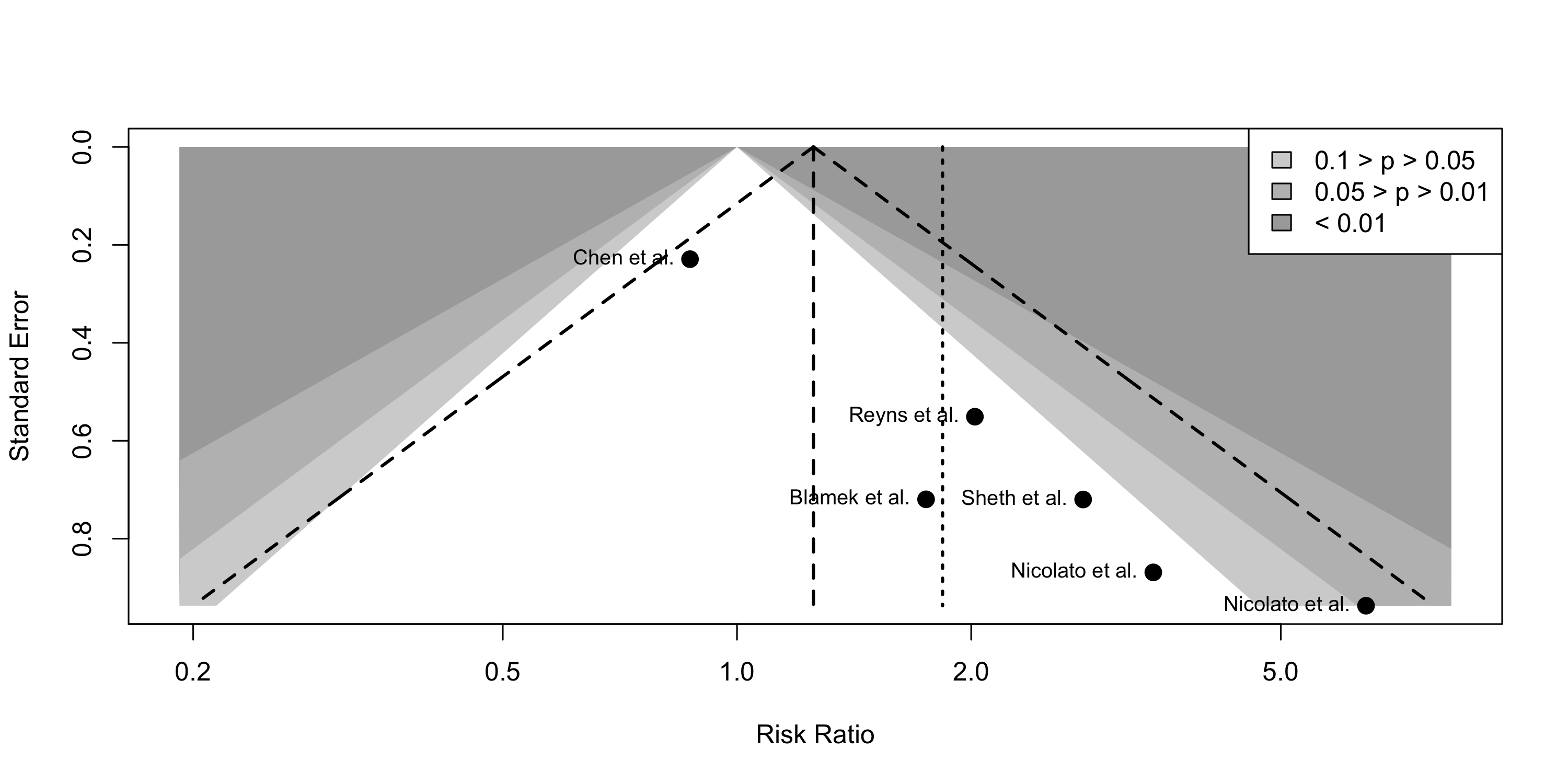


Supplemental Figure 3. Funnel Plot indicating risk of publication bias in SM grade analysis in Figure 4. t=7.79, df=4, p=0.0015, bias estimate=2.2833 (SE=0.2931).


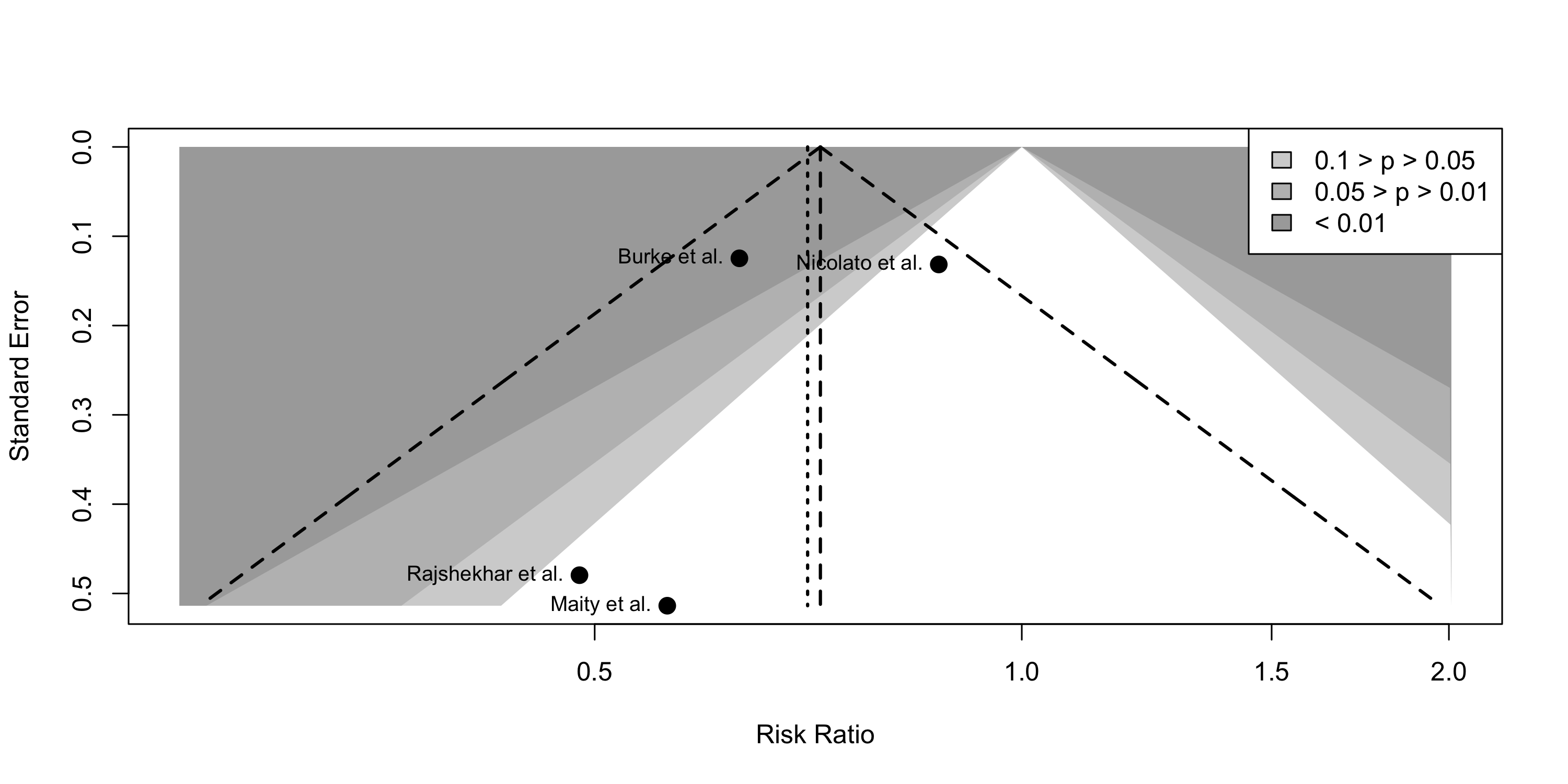


Supplemental Figure 4. Funnel Plot indicating risk of publication bias in prior procedure analysis in Figure 5. t=-0.68, df=2, p=0.5683, bias estimate=-0.8639 (SE=1.2764)
